# The fracture predictive ability of lumbar spine BMD and TBS as calculated based on different combinations of the lumbar spine vertebrae

**DOI:** 10.1007/s11657-022-01123-8

**Published:** 2022-06-09

**Authors:** Enisa Shevroja, François Mo Costabella, Elena Gonzalez Rodriguez, Olivier Lamy, Didier Hans

**Affiliations:** Interdisciplinary Center of Bone Diseases, Bone and Joint Department, Lausanne University Hospital, Lausanne, Switzerland

**Keywords:** DXA, Bone mineral density, Trabecular bone score, Fracture risk assessment, Osteoporosis

## Abstract

**Summary:**

Lumbar spine bone mineral density (BMD) and trabecular bone score (TBS) are both calculated on L1-L4 vertebrae. This study investigated the ability to predict osteoporotic fractures of BMD and TBS as calculated based on all possible adjacent L1-L4 vertebrae combinations. Present findings indicate that L1-L3 is an optimal combination to calculate LS-BMD or TBS.

**Introduction:**

Lumbar spine (LS) BMD and TBS are both assessed in the LS DXA scans in the same region of interest, L1-L4. We aimed to investigate the ability to predict osteoporotic fractures of all the possible adjacent LS vertebrae combinations used to calculate BMD and TBS and to evaluate if any of these combinations performs better at osteoporotic fracture prediction than the traditional L1-L4 combination.

**Methods:**

This study was embedded in OsteoLaus-women cohort in Switzerland. LS-DXA scans were performed using Discovery A System (Hologic). The incident vertebral fractures (VFs) and major osteoporotic fractures (MOFs) were assessed from VF assessments using Genant’s method or questionnaires (non-VF MOF). We ran logistic models using TBS and BMD to predict MOF, VF, and non-VF MOF, combining different adjustment factors (age, fracture level, or BMD).

**Results:**

One thousand six hundred thirty-two women (mean ± SD) 64.4 ± 7.5 years, BMI 25.9 ± 4.5 kg/m^2^, were followed for 4.4 years and 133 experienced MOF. The association of one SD decrease L1-L3 BMD with the odds ratios (ORs) of MOF was OR 1.32 (95%CI 1.15–1.53), L2-L4 BMD was 1.25 (95%CI 1.09–1.42), and L1-L4 BMD was 1.30 (95%CI 1.14–1.48). One SD decrease in L1-L3 TBS was more strongly associated with the odds of having a MOF (OR 1.64, 95% CI 1.34–2.00), than one SD decrease in L2-L4 TBS (OR 1.48, 95% CI 1.21–1.81), or in L1-L4 TBS (OR 1.60, CI 95% 1.32–1.95).

**Conclusion:**

Current findings indicate that L1-L3 is an optimal combination for the TBS or LS-BMD calculation.

**Supplementary Information:**

The online version contains supplementary material available at 10.1007/s11657-022-01123-8.

## Introduction

The trabecular bone score (TBS) algorithm uses the 2-dimensional (2D) projection of dual-energy X-ray absorptiometry (DXA) images to estimate the actual 3-dimensional (3D) bone structure. It measures the rate of local variations in gray levels in the 2D DXA image, which reflect the global variations in X-ray absorption properties that exist in the corresponding 3D tissue microarchitecture [1]. The correlations of TBS with bone microarchitecture parameters such as trabeculae number, trabecular thickness, connectivity, or spacing have been shown in previous studies [1–4], making the TBS a surrogate to evaluate the quality of trabecular microarchitecture. Studies have robustly shown that TBS discriminates and predicts osteoporotic fractures independently of bone mineral density (BMD) and clinical risk factors (CRFs) [5, 6]. It has therefore been incorporated into the Fracture Risk Assessment Tool (FRAX) as one of the risk factors for having an osteoporotic fracture [7].

**Fig. 1:**
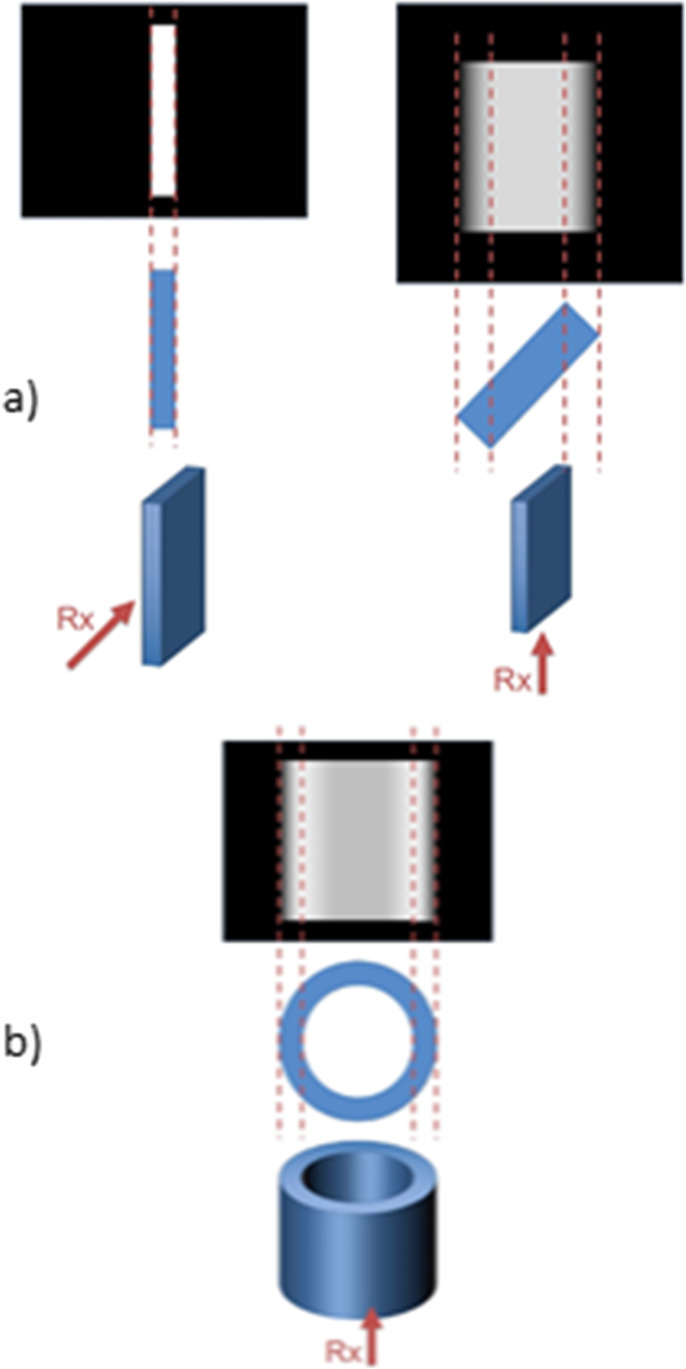
The effect of varying projection angle on the bone mineral density (**a**) and trabecular bone score (**b**) assessments from the 2D plan scans

TBS is assessed from the lumbar spine (LS) DXA scans in the same region of interest as BMD, L1-L4 vertebrae. BMD and TBS are both calculated respecting the vertebrae exclusion criteria as defined by the International Society for Clinical Densitometry (ISCD) [8]. Both scores are dependent of the positioning of the vertebrae in space. Regarding BMD, if the inclination of a vertebra varies, the area projected on a 2D surface might be altered (Fig. 1a), as for every non-spherical object. As bone mineral content (BMC) remains the same, the BMD (BMC/area) may eventually vary [9]. Regarding TBS, a positional variation, due to the erroneous positioning of the individual during the DXA acquisition or the vertebrae (lumbar lordosis), may affect the variations of gray levels in the 2D projection of the bone texture that it assesses (Fig. 1b).

We hypothesized that the lower lumbar spine vertebrae might typically be more affected by the erroneous positioning of the patient and/or the vertebrae, and their exclusion from the TBS and BMD calculations might present a more optimal estimation of both these parameters. The purpose of this study was to investigate the ability to predict osteoporotic fractures of all the possible adjacent lumbar spine vertebrae combinations used to calculate BMD and TBS from the L1-L4 DXA scans, and to evaluate if any of these combinations performs better at osteoporotic fracture prediction than the traditional L1-L4 combination.

## Methods

### Study population

The present study was embedded within the OsteoLaus cohort. Detailed information about the OsteoLaus cohort can be found elsewhere [10]. Briefly, the OsteoLaus cohort includes nearly 1500 postmenopausal women aged 50–80 years living in Lausanne, Switzerland. Baseline data were collected between March 2010 and December 2012; thereafter, follow-up visits have been conducted every 2.5 years. All the visits took place at the Interdisciplinary Center of Bone Diseases at the Lausanne University Hospital, Switzerland. At each visit, each individual underwent physical examination; LS, hip and total body DXA scans; and vertebral fracture assessment (VFA) and had a blinded calculation of TBS values. Data from the baseline, second, and third visits, comprising a mean follow-up period of 4.4 years, were used for the current analysis.

The OsteoLaus Study has been approved by the Ethics Committee for human research of Canton Vaud. All participants gave their written informed consent after having received a detailed description of the objective and funding of the study.

### Assessment of TBS and BMD in the lumbar spine

LS DXA scans were performed using Discovery A System (Hologic, 123 Waltham, MA, USA) at the baseline visit. The scanner was calibrated daily using a standard calibration block supplied by the manufacturer. All metal items were removed before densitometry, and women were examined wearing only underwear and a cloth gown. BMC in grams, area in cm^2^, and BMD in g/cm^2^ were recorded for each of the L1 to L4 vertebrae. A blind central processing (one expert validated the TBS values and another expert validated the BMD values) of TBS (TBS iNsight® 4.0, Medimaps Group, Plan-les-Ouates, Geneva, Switzerland) was performed on the LS DXA scans. In this analysis, we studied LS BMD and TBS as calculated including these vertebrae: L1-L4 (the widely clinically used combination), L1L2, L2L3, L1-L3, L2L4, and L3L4. The exclusion criteria for the vertebrae based on the ISCD guidelines were more than one standard deviation (SD) difference in BMD versus the vertebrae immediately adjacent, fractured vertebra in the scan field, LS images unreadable at that level because of severe deformations or osteosynthesis materials, vertebra with cementoplasty or hardware from surgery, or any obvious abnormalities identifiable given the resolution of the system. BMD of each combination was calculated as the ratio of the BMC of the included vertebrae with the area of the included vertebrae. LS TBS of each combination was calculated as the mean value of the individual TBS of each included vertebra. The BMD T-scores were calculated using the revised National Health and Nutrition Examination Survey (NHANES) III white female reference values for each combination being studied.

### Other covariates

Weight, height, and body mass index (BMI) were measured by the study nurse during the baseline visit. Femoral neck BMD and total hip BMD were assessed from the hip DXA scans performed using Discovery A System (Hologic, 123 Waltham, MA, USA) at the baseline visit.

### Assessment of incident fractures

For this study, longitudinal records were assessed between the baseline and the second follow-up visit (mean follow-up time: 4.4 years) for the presence of non-traumatic fractures. Vertebral fractures (VFs), major osteoporotic fractures (MOFs) (hip, VFs grade 2 or 3, forearm, and humerus fractures), or the non-VF MOFs (hip, forearm, and humerus) that occurred after the baseline visit until the second follow-up visit were the outcomes of interest. Data on the incident hip, forearm, and humerus fractures were self-reported in the questionnaires performed at each study visit, whereas the incident radiological VFs were assessed from VFA. VFA was performed for the levels T4-L4 using lateral single-energy absorptiometry images of the thoracolumbar spine on Discovery A System (Hologic, 123 Waltham, MA, USA) at the baseline and first follow-up visit and Lunar iDXA (GE Healthcare, Madison, WI, USA) at the second follow-up visit. Each reading, to determine if a VF was present or absent, was initially visual and qualitative, then VFs were classified following the semi-quantitative method developed by Genant et al. [11]. The incident VFs that were present at the first follow-up visit’s reading but not at the second follow-up visit’s reading were reevaluated by two expert readers, and in most of the cases, the reading from the second follow-up visit was considered the ultimate one due to the better VFA image quality. Further details on the VFA assessments for the OsteoLaus Study baseline and first follow-up visits may be found elsewhere [12].

### Statistical analysis

Independent-samples *t* tests were performed to assess the differences in baseline characteristics between the women who had an incident MOF, VF, or non-VF MOF during the follow-up period and those who did not. We studied the performance of each of the TBS or LS BMD combinations in VF, MOF, and non-VF MOF risk prediction. Binary logistic regression models were used to obtain the risk estimates for VFs, MOFs, or non-VF MOFs per SD decrease in LS BMD as calculated for L1, L2, L3, L4, L1L2, L2L3, L1-L3, L2-L4, L3L4, and L1-L4 in logistic models adjusted for age. Additionally, the VF and MOF models were adjusted for the VF level (at L1, L2, L3, L4 (if one fracture had occurred) or L1L2, L1L3, L1L4, or L3L4 (if two fractures had occurred)). Binary logistic regression models were used to obtain the risk estimates for MOFs, VFs, or non-VF MOFs per SD decrease in LS TBS as calculated for L1, L2, L3, L4, L1L2, L2L3, L1-L3, L2-L4, L3L4, and L1-L4 in two logistic models adjusted for (a) age and (b) age and LS BMD. In addition to age or age and LS BMD, the VF and MOF models were adjusted for the VF level (at L1, L2, L3, L4 (if one fracture had occurred) or L1L2, L1L3, L1L4, L2L3, or L3L4 (if two fractures had occurred)). Furthermore, the area under the receiver-operating-characteristic curve (AUC) was calculated for each model. The analysis using L1, L2, L3, or L4 BMD or TBS was performed for the purpose of this study solely, to have an overview of how they differ among the lumbar levels. BMD and TBS as calculated on a sole lumbar vertebra are not clinically meaningful. 2 × 2 contigency tables were used to calculate the specificity and precision of BMD and TBS as calculated on the L1-L4 or L1-L3. After adjusting for multiple comparisons, *p* < 0.005 was set as the level of statistical significance. Statistical analyses were performed by using SPSS 25.0 (SPSS Inc., Chicago, IL, USA).

## Results

**Fig. 2:**
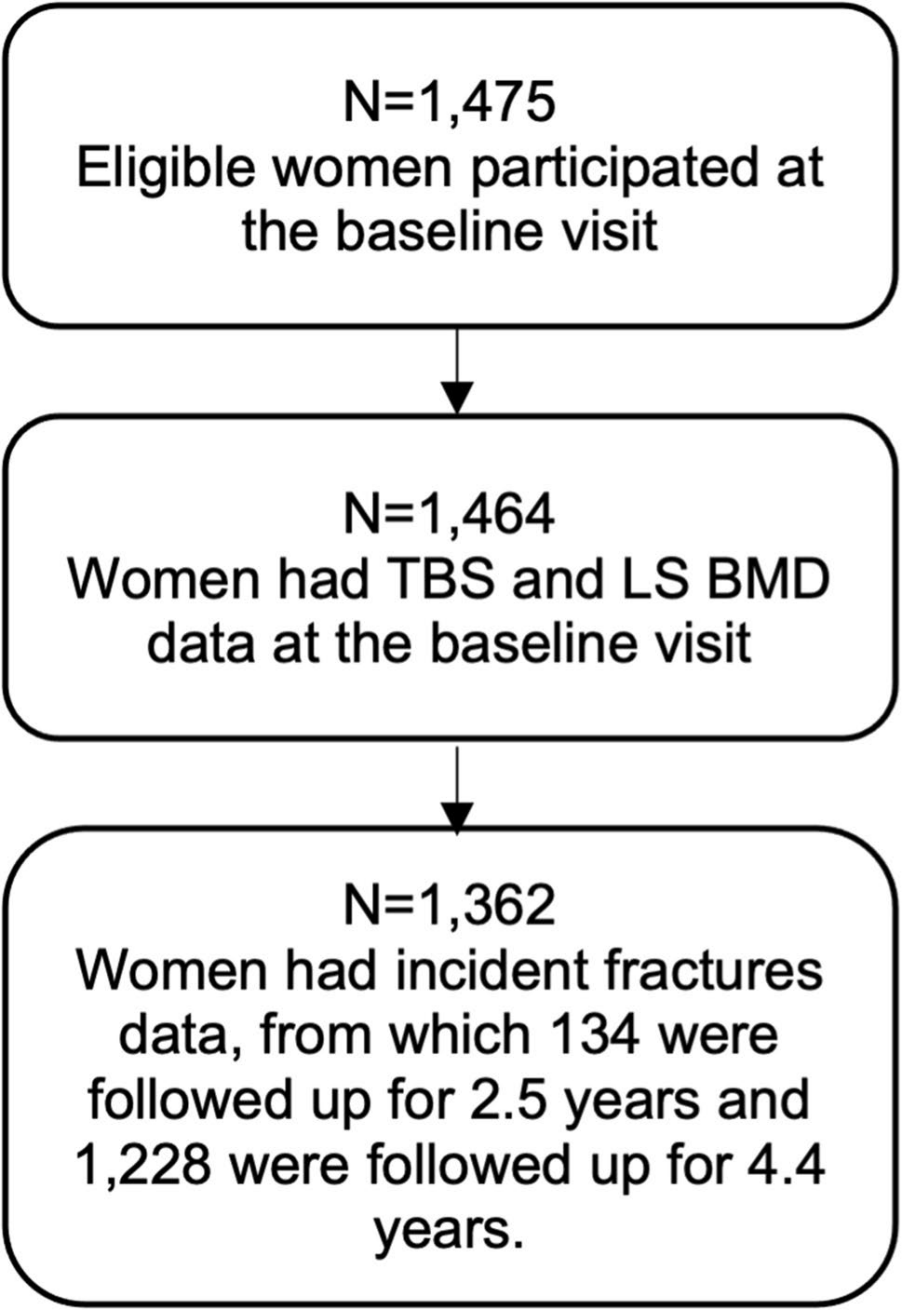
Flowchart of the study population

In this analysis, 1362 women who participated in the OsteoLaus baseline visit and had data on incident MOF either at first, second, or both follow-up visits were included. A flowchart of the study population is shown in Fig. 2. The baseline general characteristics of the study participants are shown in Table 1. The age of the participants was (mean ± SD) 64.4 ± 7.5 years; BMI 25.9 ± 4.5 kg/m^2^; LS BMD T-score − 1.04 ± 1.47 SD and TBS 1.322 ± 0.100. In total, 53 participants had a prevalent vertebral fracture at the baseline visit. From these, in the lumbar vertebrae level, 13 were in L1, 4 in L2, 2 in L3, and 0 in L4.

**Table 1:** Baseline general characteristics of the study participants

	All participants (*N* = 1362)	MOF		VF		Non-VF MOF	
		Yes (*n* = 133)	No (*n* = 1229)	Yes (*n* = 87)	No (*n* = 1275)	Yes (*n* = 46)	No (*n* = 1316)
Age (years)	64.4 (7.5)	68.1 (7.3)	64.0 (7.4)*	68.3 (7.5)	64.1 (7.4)*	67.7 (7.0)	64.3 (7.5)*
Weight (kg)	67.4 (12.0)	68.9 (12.3)	67.2 (12.0)	67.9 (12.1)	67.4 (12.0)	70.7 (12.5)	67.3 (12.0)
Height (cm)	161.4 (6.6)	161.7 (7.3)	161.4 (6.6)	161.6 (7.4)	161.4 (6.6)	161.8 (7.2)	161.4 (6.6)
BMI (kg/m^2^)	25.9 (4.5)	26.3 (4.3)	25.8 (4.5)	26.0 (4.3)	25.9 (4.5)	27.0 (4.3)	25.9 (4.5)
L1-L4 TBS (unitless)	1.32 (0.10)	1.27 (0.10)	1.33 (0.10)*	1.27 (0.10)	1.33 (0.10)*	1.28 (0.09)	1.32 (0.10)*
L1-L4 BMD T-score (SD)	− 1.04 (1.47)	− 1.53 (1.46)	− 0.99 (1.46)*	− 1.65 (1.51)	− 1.00 (1.46)*	− 1.29 (1.33)	− 1.03 (1.47)
FN BMD T-score (SD)	− 1.07 (1.03)	− 1.46 (0.93)	− 1.03 (1.03)*	− 1.52 (0.99)	− 1.04 (1.03)*	− 1.35 (0.80)	− 1.05 (1.04)
Total hip BMD T-score (SD)	− 0.71 (0.96)	− 1.15 (0.94)	− 0.66 (0.95)*	− 1.24 (1.0)	− 0.67 (0.95)*	− 0.99 (0.81)	− 0.69 (0.96)*

Values are mean (standard deviation) unless indicated otherwise

*MOF* major osteoporotic fracture, *VF* vertebral fracture, *BMI* body mass index, *TBS* trabecular bone score, *BMD* bone mineral density, *FN* femoral neck

^*^Significant at the 0.05 level

As based on the ISCD vertebrae exclusion criteria, from the lumbar spine, 24 L1 (13 of which were due to fracture presence), 219 L2 (4 of which were due to fracture presence), 233 L3 (2 of which were due to fracture presence), and 183 L4 (0 fractured) were excluded at baseline. The individuals who had at least one excluded lumbar vertebrae due to these criteria were older and had higher BMI values than those who had no lumbar vertebrae excluded. The follow-up time did not change among them.

During the mean follow-up period of 4.4 years, 133 women experienced a MOF. From these women, nine had two MOFs, from which four had one forearm and one vertebral fracture; two had one forearm and one humerus fracture; two had one hip and one vertebral fracture; and one woman had one humerus and one vertebral fracture; all others had only one MOF. Among MOF, seven were hip fractures, 87 vertebral, 33 forearm, and 15 humerus fractures. From the 87 incident vertebral fractures, 11 had also had another vertebral fracture previously. Those who fractured were older; had lower BMD at the LS, FN, and hip; lower TBS; and higher FRAX values than those who did not fracture.

In Tables 2 and 3, we show the odds ratios of having a fracture per each SD decrease in LS BMD and in TBS as calculated per each vertebrae combination being studied, respectively. The corresponding AUC values for each model are shown in Supplementary Material tables S1 and S2. In overall, based on the observed OR values, we see a tendency of the L4 BMD and L4 TBS to be more poorly associated with the risk of having a fragility fracture than the other upper vertebrae. In the models adjusted for age, the odds of having a MOF increased by 18% (OR 1.18, 95% CI 1.07–1.31) for one SD decrease in L4 BMD, and 32% (OR 1.32, 95% CI 1.09–1.60) for one SD decrease of L4 TBS, whereas for one SD decrease in L1 BMD and L1 TBS, the odds of having a MOF increased 35% (OR 1.35, 95% CI 1.16–1.57) and 63% (OR 1.63, 95% CI 1.35–1.96), respectively. A similar tendency was seen in the other models as well. Furthermore, one SD decrease in LS BMD and TBS calculated from the combinations of lower lumbar vertebrae (typically L3, L4) was more poorly associated with the odds ratio of having a fracture than one SD decrease in LS BMD and LS TBS calculated from the combinations of upper lumbar vertebrae (typically L1, L2), as based on the observed OR values.

**Table 2:** Odds ratio of having a fracture per each SD decrease in LS BMD

Model’s covariates	LS vertebrae included in BMD calculation	OR (95% CI) per each SD decrease in LS BMD		
		MOF	VF	Non-VF MOF
Age	L1	1.35 (1.16–1.56)	1.41 (1.17–1.69)	1.19 (0.95–1.50)
	L2	1.25 (1.10–1.42)	1.35 (1.15–1.58)	1.07 (0.88–1.30)
	L3	1.22 (1.09–1.37)	1.28 (1.11–1.47)	1.10 (0.93–1.31)
	L4	1.18 (1.06–1.31)	1.21 (1.07–1.38)	1.11 (0.94–1.31)
	L1L2	1.41 (1.19–1.68)	1.43 (1.16–1.76)	1.31 (0.99–1.75)
	L2L3	1.34 (1.13–1.59)	1.33 (1.08–1.63)	1.28 (0.97–1.69)
	L1-L3	1.32 (1.15–1.53)	1.40 (1.17–1.68)	1.15 (0.92–1.44)
	L2-L4	1.25 (1.09–1.42)	1.30 (1.11–1.52)	1.12 (0.91–1.37)
	L3L4	1.26 (1.08–1.46)	1.41 (1.17–1.71)	1.00 (0.80–1.25)
	L1-L4	1.30 (1.14–1.48)	1.38 (1.17–1.62)	1.12 (0.92–1.38)
Age + VF_level	L1	1.30 (1.11–1.52)	1.33 (1.10–1.63)	
	L2	1.20 (1.05–1.37)	1.28 (1.07–1.53)	
	L3	1.18 (1.05–1.33)	1.23 (1.05–1.43)	
	L4	1.15 (1.03–1.28)	1.15 (1.01–1.32)	
	L1L2	1.33 (1.10–1.60)	1.29 (1.02–1.63)	
	L2L3	1.31 (1.09–1.57)	1.29 (1.03–1.62)	
	L1-L3	1.24 (1.07–1.44)	1.27 (1.05–1.54)	
	L2-L4	1.17 (1.02–1.34)	1.17 (0.99–1.39)	
	L3L4	1.17 (1.00–1.37)	1.29 (1.05–1.58)	
	L1-L4	1.23 (1.07–1.41)	1.28 (1.07–1.53)	

*SD* standard deviation, *LS* lumbar spine, *BMD* bone mineral density, *MOF* major osteoporotic fracture, *VF* vertebral fracture, *OR* odds ratio, *CI* confidence interval, *TBS* trabecular bone score

**Table 3:** Odds ratio of having a fracture per each SD decrease in TBS

Model’s covariates	LS vertebrae included in TBS calculation	OR (95% CI) per each SD decrease in TBS		
		MOF	VF	Non-VF MOF
Age	L1	1.63 (1.35–1.96)	1.79 (1.43–2.24)	1.26 (0.94–1.69)
	L2	1.52 (1.26–1.84)	1.56 (1.24–1.96)	1.35 (1.00–1.82)
	L3	1.40 (1.16–1.68)	1.42 (1.14–1.77)	1.29 (0.96–1.74)
	L4	1.32 (1.09–1.59)	1.29 (1.03–1.62)	1.32 (0.98–1.80)
	L1L2	1.77 (1.42–2.21)	1.78 (1.37–2.31)	1.58 (1.11–2.26)
	L2L3	1.56 (1.23–1. 96)	1.47 (1.12–1.94)	1.59 (1.09–2.32)
	L1-L3	1.64 (1.34–2.00)	1.70 (1.34–2.16)	1.41 (1.03–1.92)
	L2-L4	1.48 (1.21–1.81)	1.47 (1.16–1.87)	1.41 (1.02–1.94)
	L3L4	1.44 (1.15–1.81)	1.56 (1.18–2.05)	1.18 (0.82–1.70)
	L1-L4	1.60 (1.32–1.95)	1.68 (1.32–2.13)	1.36 (1.00–1.85)
Age + VF_level	L1	1.57 (1.29–1.92)	1.74 (1.35–2.24)	
	L2	1.45 (1.18–1.77)	1.46 (1.13–1.89)	
	L3	1.31 (1.07–1.60)	1.28 (0.99–1.65)	
	L4	1.24 (1.02–1.51)	1.16 (0.90–1.49)	
	L1L2	1.68 (1.32–2.13)	1.64 (1.21–2.22)	
	L2L3	1.51 (1.18–1.93)	1.39 (1.02–1.90)	
	L1-L3	1.52 (1.23–1.88)	1.52 (1.16–1.99)	
	L2-L4	1.34 (1.09–1.66)	1.25 (0.95–1.63)	
	L3L4	1.28 (1.00–1.63)	1.30 (0.96–1.77)	
	L1-L4	1.48 (1.20–1.82)	1.50 (1.15–1.96)	
Age + LS BMD	L1	1.49 (1.18–1.87)	1.64 (1.25–2.16)	1.16 (0.80–1.67)
	L2	1.39 (1.10–1.76)	1.33 (1.00–1.76	1.43 (0.98–2.07)
	L3	1.24 (0.99–1.54)	1.20 (0.92–1.56)	1.27 (0.89–1.81)
	L4	1.17 (0.94–1.46)	1.10 (0.84–1.43)	1.28 (0.89–1.83)
	L1L2	1.62 (1.22–2.15)	1.62 (1.15–2.26)	1.49 (0.94–2.37)
	L2L3	1.37 (1.03–1.82)	1.27 (0.91–1.79)	1.49 (0.94–2.37)
	L1-L3	1.51 (1.16–1.95)	1.48 (1.08–2.02)	1.44 (0.95–2.18)
	L2-L4	1.35 (1.04–1.74)	1.25 (0.92–1.69)	1.47 (0.97–2.22)
	L3L4	1.28 (0.97–1.68)	1.24 (0.89–1.73)	1.29 (0.83–2.00)
	L1-L4	1.46 (1.12–1.89)	1.43 (1.05–1.96)	1.40 (0.93–2.12)
Age + LS BMD + VF_level	L1	1.46 (1.15–1.87)	1.67 (1.22–2.28)	
	L2	1.37 (1.07–1.75)	1.28 (0.93–1.76)	
	L3	1.17 (0.92–1.49)	1.08 (0.80–1.46)	
	L4	1.12 (0.88–1.41)	1.01 (0.75–1.36)	
	L1L2	1.61 (1.18–2.18)	1.61 (1.09–2.39)	
	L2L3	1.34 (0.98–1.82)	1.20 (0.81–1.78)	
	L1-L3	1.46 (1.11–1.93)	1.42 (0.99–2.02)	
	L2-L4	1.27 (0.97–1.67)	1.11 (0.78–1.58)	
	L3L4	1.17 (0.87–1.57)	1.06 (0.73–1.55)	
	L1-L4	1.38 (1.05–1.82)	1.32 (0.93–1.89)	

*SD* standard deviation, *TBS* trabecular bone score, *MOF* major osteoporotic fracture, *VF* vertebral fracture, *OR* odds ratio, *CI* confidence interval, *LS* lumbar spine, *BMD* bone mineral density

### BMD calculated with different LS vertebrae combinations and fracture risk

One SD decrease in BMD as calculated based on three or four vertebrae combinations, namely, L1-L3, L2-L4, L1-L4, was slightly more strongly associated with the odds ratios of having a VF than with the odds ratios of having a MOF, as observed from their OR values. Among these three combinations, one SD decrease in L1-L3 BMD was more strongly associated with the odds of having a VF (OR 1.40 95%CI 1.17–1.68) or MOF (OR 1.32 95%CI 1.15–1.53) and one SD decrease L2-L4 BMD more weakly (VF (OR 1.30 95%CI 1.11–1.52) or MOF (OR 1.25 95%CI 1.09–1.42)). The association of one SD decrease L1-L4 BMD with the odds ratios of VF was OR 1.38 (95%CI 1.17–1.62) and MOF was 1.30 (95%CI 1.14–1.48). This tendency was present after adjusting for the vertebral fracture level. All three combinations were more poorly (lower OR values) and not statistically significantly associated with the risk of having a non-VF MOF. For example, the association of one SD decrease L1-L3 BMD with the odds ratios of non-VF MOF was OR 1.15 (95%CI 0.92–1.44) and of one SD decrease L2-L4 BMD was 1.00 (95%CI 0.80–1.25).

The specificity of L1-L4 BMD in incident MOF discrimination was 88%, and its precision was 16%. Similarly, for L1-L3 BMD, the specificity and precision were 90% and 16%, respectively. Regarding the reclassification of individuals from using the classical L1-L4 BMD to the L1-L3 BMD, we see that L1-L3 BMD would reclassify 31% of L1-L4-BMD-based osteoporotics as osteopenic, 15% of L1-L4-BMD-based osteopenic as normal, and 99% of L1-L4-BMD-based normals as normal.

### TBS calculated with different LS vertebrae combinations and fracture risk

In general, TBS showed a better performance than BMD in fracture risk prediction when used on the different vertebrae combinations, as one SD decrease in TBS was associated with higher odds of having a fracture of any type (MOF, VF, and non-VF MOF) than one SD decrease in LS BMD. For example, the best performing combinations, L1-L3, in the model adjusted for age, were associated with 32% (OR 1.32, 95% CI 1.15–1.53) increase in the odds of having a MOF for one SD decrease in L1-L3 BMD and 64% (OR 1.64, 95% CI 1.34–2.00) for one SD decrease in L1-L3 TBS. A similar tendency was observed in BMD and TBS calculated based on other combinations and in the other models. In general, the additional model’s adjustment for BMD slightly weakened the association of TBS with the odds of fractures. Similarly as for BMD, among the three vertebrae combinations (L1-L3, L2-L4, and L1-L4) for TBS, the weakest association with MOF and VF was found with the L2-L4 TBS and the strongest with L1-L3 TBS, as based on the OR values. For example, in the models adjusted for age, one SD decrease in L1-L3 TBS was the most strongly associated with the odds of having a MOF (OR 1.64, 95% CI 1.34–2.00), one SD decrease in L2-L4 TBS was the most weakly associated (OR 1.48, 95% CI 1.21–1.81), and L1-L4 TBS lied in between (OR 1.60, CI 95% 1.32–1.95). A similar tendency among these three combinations was observed in the models additionally adjusted for BMD, VF level, or both, for MOF or VF. This tendency was not present in the non-VF MOF analysis, yet the results were not statistically significant. The specificity of L1-L4 TBS in incident MOF discrimination was 58%, and its precision was 15%. Similarly, for L1-L3 TBS, the specificity and precision were 59% and 16%, respectively.

## Discussion

In this study of Swiss postmenopausal women, we observed that the LS BMD and TBS of the more highly positioned lumbar vertebrae and their combinations were better predictors of incident fracture as compared to the lower vertebrae. More specifically, the exclusion of L4 and the inclusion of L1 in the calculation of LS BMD and TBS were associated with higher odds of having an incident fracture.

In clinical practice, the calculation of LS BMD and TBS cannot be based on a single lumbar vertebra; we show these here solely for the purpose of tendency’s observation. Also, the use of LS BMD and TBS values calculated based on two vertebrae is highly risky to be suggested for clinical routine use, as the likelihood of excluding one vertebra following the ISCD recommendations is high. They were also shown here for observation purposes. Our findings suggest that both BMD and TBS calculated on L1L2 perform better than any other combination at the association with the odds of having a fracture. However, we do not elaborate further on this combination because, as stated above, it is clinically risky to recommend the use of only two vertebrae to calculate LS BMD and TBS. LS BMD and TBS calculated as based on the combinations of three or four lumbar vertebrae, L1-L3, L2-L4, and L1-L4, are clinically relevant. Among these three combinations, we saw that L1-L3 had the strongest association with odds of future fracture and L2-L4 the weakest. This finding is in accordance with what was observed on the individual vertebrae, where L1 had the strongest association with odds of fracture and L4 the weakest. Namely, the inclusion of L1 and the exclusion of L4 from the LS BMD and TBS calculations seemed to improve their ability to predict fractures.

Multiple studies have shown that the frequency of fractures in the lumbar spine decreases with the vertebra level [13–16], with L1 having the highest frequency of fracture occurrence. Its position in the thoracolumbar junction exposes L1 to the compressive forces applied mainly during the spinal flexion, making it more prone to fractures [16]. This fact enables the assumption that LS BMD and TBS calculated based on a vertebrae combination including L1 are better predictors of future VF and eventually MOF. To address this issue, our models were additionally adjusted for the level of the vertebra where the incident VF happened. After this adjustment, lower OR values than in the models unadjusted for the fracture level were obtained. However, the tendency remained the same: L1-L3 BMD and TBS had the strongest association with the odds of having a VF or MOF, and L2-L4 had the weakest. Simultaneously, the adjustment for the VF level addresses the assumption that LS BMD and TBS calculated based on a vertebrae combination including L4 are poorer fracture predictors due to the fact that less fractures occur at L4 [13–16].

**Fig. 3:**
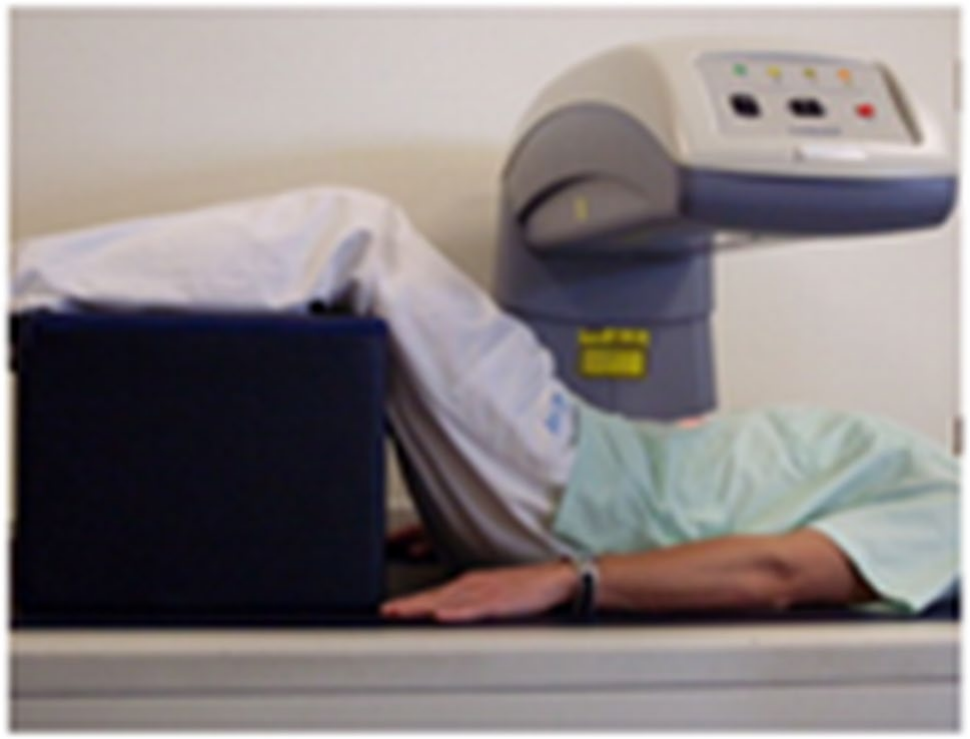
Patient’s positioning on the DXA machine to flatten the lumbar lordosis

We may speculate that the lower association with fracture odds of L4 BMD and TBS as compared to the BMD and TBS of the higher lumbar levels — L1, for example — is due to a positioning issue of the individual during LS DXA acquisition. The spine naturally forms an inward curvature in the lumbar region, namely, the lumbar lordosis. Thus, L1-L4 are not spontaneously parallel to the plan of the DXA machine. For an accurate LS DXA acquisition, this lordosis has to be flattened by a hip flexion, facilitated by positioning the legs on a padded box with a fixed height (Fig. 3) [17]. In consequence, individuals of different sizes, particularly with different femur lengths, will have different hip flexion angles. The more the hip flexion angle tends to 0°, the more lordosis will be present during the image acquisition. A good positioning during the DXA acquisition is crucial for the accuracy of this test. However, multiple studies have shown that incorrect positioning is a frequent problem [18–21]. DXA technologists of our center have received training to avoid these errors, a practice proven efficient to greatly minimize them [22]. Nevertheless, the minimization techniques might not completely eliminate the incorrect positioning effect on the DXA image. This erroneous positioning of the vertebrae affects the projected vertebrae surface on the 2D DXA image. Both BMD, which is calculated as BMC/area, and TBS, which is calculated based on the gray levels’ variations in the 2D DXA image, are simultaneously affected. This explains the similar tendency seen in the investigation of both LS BMD and TBS.

Furthermore, degenerative disease (DD), such as osteoarthritis, is more present in the L4 vertebra as compared to L1-L3 [23]. Its presence increases the values of BMD artefactually. To address this issue, one of the ISCD recommendations to exclude the vertebrae with a BMD T-score exceeding 1 SD from the adjacent vertebra is applied. However, following this recommendation does not eliminate the presence of DD in the vertebra entirely, in cases where the artefactually increased BMD values have not reached the threshold implied by this recommendation. On the contrary, the TBS value is not affected by the DD changes in the vertebrae [23].

Relying on three vertebrae instead of the currently four vertebrae approach (L1-L4) for the LS BMD and TBS calculations presents uncertainty given the vertebrae exclusion rules (L1-L3 is less affected than L4, but not unaffected) and the fact that LS BMD and TBS cannot be calculated in only one lumbar vertebra. However, in Japan, guidelines propose the use of three vertebrae, L2-L4, to calculate LS BMD, indicating that it is actually possible in clinical practice [24]. Nevertheless, this combination might jeopardize fracture risk by underestimating it. We would strongly suggest the inclusion of L1 in the vertebrae combination chosen to calculate TBS or LS BMD, given L1 seems to be less affected from the above stated factors.

Certain limitations are present in our study. Firstly, our population includes only postmenopausal women, who are for the vast majority (98,4%) Caucasian. It is yet to be proven that our results apply in men or other ethnicities. Nonetheless, BMD was proven to be as good a predictor of fracture among men as among women [25, 26], and the same has been shown for TBS [5]. BMD performance varies significantly by race in postmenopausal women [27], and TBS showed a lower performance in fracture risk prediction in African American population than in Caucasian [28]. Secondly, the number of participants used for each combination`s analysis, was not the same, as individuals were also subject to lumbar vertebrae exclusion based on the ISCD criteria. However, 83% of the participants overlapped at each analysis. Thirdly, the number of incident fractures that occurred during the follow-up period in our cohort is limited. Larger studies would give broader insights and elaborate further our observations. Lastly, our data were collected on Hologic machines. Studies show that significant variations in measurements happen between different models of a same manufacturer [29], and even between two copies of the same model [30]. Our results are yet to be reproduced on devices of other manufacturer.

In conclusion, our findings suggest that the exclusion of L4 and the inclusion of L1 in general in the LS BMD and TBS calculations improve their performance in fracture risk prediction. This can be explained by the fact that the lower-level lumbar vertebrae might be more exposed to erroneous positioning of the individual during the LS DXA acquisition and of the degenerative disease`s presence. We are limited to suggest the use of L1-L3 — which is the combination appearing more promising from our findings — instead of the L1-L4 because further investigations of its specificity, precision, and sensitivity in fracture prediction would be needed to support such recommendation. This effort needs to be replicated in larger studies’ settings to give assertive clinical recommendations.

## Supplementary Information

Below is the link to the electronic supplementary material.

Supplementary file1 (DOCX 22 KB)
