# Supplementary material for: The fracture predictive ability of lumbar spine BMD and TBS as calculated based on different combinations of the lumbar spine vertebrae
Source: Arch Osteoporos. 2022 Jun 9;17(1):83. doi: 10.1007/s11657-022-01123-8 (PMC9184435; doi:10.1007/s11657-022-01123-8)
Supplement: Supplementary file 1 — Supplementary file1 (DOCX 22 KB) [file 11657_2022_1123_MOESM1_ESM.docx]

**Supplementary Material**

**Table S1.** AUC values of the logistic models whete the exposure of interest was LS BMD as calculated based on the different lumbar spine vertebrae combinations

| **Model`s covariates** | **LS vertebrae included in BMD calculation** | **AUC (95% CI) of the model where the exposure is one SD decrease in LS BMD** | | |
| --- | --- | --- | --- | --- |
|  |  | **MOF** | **VF** | **Non-VF MOF** |
| **Age** | **L1** | 0.687 (0.640-0.734) | 0.693 (0.636-0.750) | 0.652 (0.574-0.730) |
|  | **L2** | 0.681 (0.634-0.727) | 0.698 (0.642-0.754) | 0.634 (0.558-0.711) |
|  | **L3** | 0.679 (0.633-0.726) | 0.688 (0.632-0.745) | 0.642 (0.565-0.718) |
|  | **L4** | 0.673 (0.626-0.721) | 0.676 (0.618-0.735) | 0.644 (0.569-0.720) |
|  | **L1L2** | 0.680 (0.627-0.733) | 0.680 (0.617-0.743) | 0.658 (0.565-0.751) |
|  | **L2L3** | 0.675 (0.620-0.730) | 0.665 (0.599-0.731) | 0.671 (0.578-0.763) |
|  | **L1-L3** | 0.687 (0.639-0.734) | 0.696 (0.638-0.754) | 0.647 (0.571-0.724) |
|  | **L2-L4** | 0.678 (0.631-0.726) | 0.682 (0.624-0.740) | 0.649 (0.572-0.726) |
|  | **L3L4** | 0.680 (0.624-0.735) | 0.696 (0.628-0.763) | 0.669 (0.583-0.755) |
|  | **L1-L4** | 0.685 (0.638-0.731) | 0.695 (0.639-0.752) | 0.642 (0.565-0.718) |
| **Age+fx_level** | **L1** | 0.754 (0.706-0.802) | 0.805 (0.748-0.862) |  |
|  | **L2** | 0.746 (0.698-0.795) | 0.804 (0.746-0.862) |  |
|  | **L3** | 0.746 (0.697-0.794) | 0.797 (0.738-0.855) |  |
|  | **L4** | 0.745 (0.697-0.793) | 0.796 (0.736-0.855) |  |
|  | **L1L2** | 0.756 (0.702-0.811) | 0.798 (0.735-0.862) |  |
|  | **L2L3** | 0.749 (0.692-0.806) | 0.783 (0.713-0.853) |  |
|  | **L1-L3** | 0.751 (0.702-0.799) | 0.801 (0.742-0.860) |  |
|  | **L2-L4** | 0.749 (0.700-0.797) | 0.796 (0.737-0.856) |  |
|  | **L3L4** | 0.742 (0.685-0.799) | 0.785 (0.714-0.856) |  |
|  | **L1-L4** | 0.749 (0.701-0.796) | 0.801 (0.743-0.858) |  |

SD = standard deviation; LS = lumbar spine; BMD = bone mineral density; MOF = major osteoporotic fracture; VF = vertebral fracture; AUC = area under the curve; CI = confidence interval; fx = fracture.

**Table S2.** AUC values of the logistic models whete the exposure of interest was TBS as calculated based on the different lumbar spine vertebrae combinations

| **Model`s covariates** | **LS vertebrae included in BMD calculation** | **AUC (95% CI) of the model where the exposure is one SD decrease in TBS** | | |
| --- | --- | --- | --- | --- |
|  |  | **MOF** | **VF** | **Non-VF MOF** |
| **Age** | **L1** | 0.697 (0.650-0.745) | 0.717 (0.660-0.775) | 0.640 (0.561-0.718) |
|  | **L2** | 0.689 (0.642-0.735) | 0.690 (0.632-0.748) | 0.663 (0.588-0.737) |
|  | **L3** | 0.672 (0.625-0.720) | 0.674 (0.615-0.732) | 0.648 (0.570-0.726) |
|  | **L4** | 0.669 (0.622-0.717) | 0.671 (0.614-0.729) | 0.645 (0.568-0.721) |
|  | **L1L2** | 0.704 (0.652-0.756) | 0.705 (0.642-0.769) | 0.678 (0.593-0.763) |
|  | **L2L3** | 0.682 (0.626-0.738) | 0.668 (0.599-0.736) | 0.689 (0.598-0.779) |
|  | **L1-L3** | 0.699 (0.651-0.747) | 0.705 (0.644-0.765) | 0.664 (0.589-0.738) |
|  | **L2-L4** | 0.686 (0.638-0.734) | 0.683 (0.624-0.743) | 0.668 (0.592-0.744) |
|  | **L3L4** | 0.681 (0.625-0.736) | 0.684 (0.615-0.753) | 0.670 (0.582-0.758) |
|  | **L1-L4** | 0.695 (0.648-0.742) | 0.704 (0.646-0.762) | 0.655 (0.579-0.731) |
| **Age+fx_level** | **L1** | 0.760 (0.713-0.807) | 0.822 (0.767-0.876) |  |
|  | **L2** | 0.757 (0.710-0.804) | 0.805 (0.748-0.863) |  |
|  | **L3** | 0.746 (0.697-0.794) | 0.797 (0.739-0.854) |  |
|  | **L4** | 0.741 (0.693-0.788) | 0.793 (0.735-0.850) |  |
|  | **L1L2** | 0.779 (0.728-0.830) | 0.822 (0.761-0.882) |  |
|  | **L2L3** | 0.761 (0.706-0.817) | 0.795 (0.727-0.863) |  |
|  | **L1-L3** | 0.761 (0.713-0.809) | 0.811 (0.753-0.869) |  |
|  | **L2-L4** | 0.753 (0.705-0.801) | 0.797 (0.738-0.856) |  |
|  | **L3L4** | 0.739 (0.683-0.796) | 0.776 (0.706-0.845) |  |
|  | **L1-L4** | 0.755 (0.708-0.802) | 0.805 (0.748-0.862) |  |
| **Age+LS BMD** | **L1** | 0.703 (0.656-0.749) | 0.720 (0.663-0.777) | 0.651 (0.573-0.729) |
|  | **L2** | 0.693 (0.646-0.740) | 0.702 (0.645-0.760) | 0.659 (0.585-0.733) |
|  | **L3** | 0.682 (0.635-0.730) | 0.691 (0.633-0.749) | 0.649 (0.570-0.727) |
|  | **L4** | 0.675 (0.628-0.723) | 0.678 (0.619-0.736) | 0.647 (0.571-0.723) |
|  | **L1L2** | 0.706 (0.654-0.758) | 0.705 (0.642-0.768) | 0.682 (0.597-0.767) |
|  | **L2L3** | 0.692 (0.637-0.747) | 0.675 (0.608-0.742) | 0.697 (0.607-0.787) |
|  | **L1-L3** | 0.701 (0.653-0.749) | 0.709 (0.649-0.769) | 0.662 (0.587-0.736) |
|  | **L2-L4** | 0.687 (0.639-0.736) | 0.688 (0.629-0.747) | 0.667 (0.591-0.744) |
|  | **L3L4** | 0.685 (0.629-0.741) | 0.701 (0.631-0.770) | 0.669 (0.581-0.758) |
|  | **L1-L4** | 0.697 (0.650-0.744) | 0.706 (0.648-0.764) | 0.654 (0.579-0.729) |
| **Age+LS BMD+lv_fx_level** | **L1** | 0.762 (0.715-0.809) | 0.821 (0.767-0.876) |  |
|  | **L2** | 0.757 (0.710-0.804) | 0.807 (0.750-0.865) |  |
|  | **L3** | 0.747 (0.699-0.796) | 0.798 (0.739-0.856) |  |
|  | **L4** | 0.743 (0.695-0.791) | 0.796 (0.736-0.855) |  |
|  | **L1L2** | 0.779 (0.728-0.830) | 0.821 (0.760-0.882) |  |
|  | **L2L3** | 0.766 (0.711-0.821) | 0.791 (0.723-0.860) |  |
|  | **L1-L3** | 0.761 (0.714-0.809) | 0.811 (0.752-0.869) |  |
|  | **L2-L4** | 0.753 (0.704-0.801) | 0.796 (0.737-0.856) |  |
|  | **L3L4** | 0.740 (0.684-0.797) | 0.784 (0.713-0.855) |  |
|  | **L1-L4** | 0.755 (0.708-0.802) | 0.804 (0.747-0.861) |  |

SD = standard deviation; TBS = trabecular bone score; MOF = major osteoporotic fracture; VF = vertebral fracture; AUC = area under the curve; CI = confidence interval; fx = fracture.
